# miR-324-3p Suppresses Hepatic Stellate Cell Activation and Hepatic Fibrosis Via Regulating SMAD4 Signaling Pathway

**DOI:** 10.1007/s12033-024-01078-w

**Published:** 2024-02-26

**Authors:** Si-Yu Chen, Xin Chen, Sai Zhu, Jin-Jin Xu, Xiao-Feng Li, Na-Na Yin, Yan-Yan Xiao, Cheng Huang, Jun Li

**Affiliations:** 1 Department of Pharmacy, Hefei BOE Hospital, Intersection of Dongfang Avenue and Wenzhong Road, Hefei, China; 2 School of Pharmacy, Anhui Medical University, 81 Mei Shan Road, Hefei, 230032 Anhui China

**Keywords:** miR-324-3p, Hepatic fibrosis, LX-2 cell activation, SMAD4

## Abstract

In hepatic fibrosis (HF), hepatic stellate cells (HSCs) form the extracellular matrix (ECM), and the pathological accumulation of ECM in the liver leads to inflammation. Our previous research found that miR-324-3p was down-regulated in culture-activated human HSCs. However, the precise effect of miR-324-3p on HF has not been elucidated. In this study, the HF mouse models were induced through directly injecting carbon tetrachloride (CCl_4_) into mice; the HF cell models were constructed using TGF-β1-treated LX-2 cells. Next, real-time-quantitative polymerase chain reaction (RT-qPCR), western blot (WB) and immunohistochemistry (IHC) were applied to assess the expression levels of miR-324-3p, α-smooth muscle actin (α-SMA), Vimentin or SMAD4; hematoxylin and eosin (H&E), Masson’ s trichrome and Sirius red staining to evaluate the liver injury; luciferase reporter assay to verify the targeting relationship between miR-324-3p and SMAD4; enzyme-linked immunosorbent assay (ELISA) to determine the levels of serum alanine aminotransferase (ALT) and aspartate aminotransferase (AST); and cell counting kit-8 (CCK-8) and flow cytometry to evaluate the effects of miR-324-3p on cell proliferation and cycle/apoptosis, respectively. The experimental results showed a reduction in miR-324-3p level in CCl_4_-induced HF mice as well as transforming growth factor (TGF)-β1-activated HSCs. Interestingly, the miR-324-3p level was rescued following the HF recovery process. In HF mice induced by CCl_4_, miR-324-3p overexpression inhibited liver tissue damage, decreased serum ALT and AST levels, and inhibited fibrosis-related biomarkers (α-SMA, Vimentin) expression, thereby inhibiting HF. Similarly, miR-324-3p overexpression up-regulated α-SMA and Vimentin levels in HF cells, while knockdown of miR-324-3p had the opposite effect. Besides, miR-324-3p played an antifibrotic role through inhibiting the proliferation of hepatocytes. Further experiments confirmed that miR-324-3p targeted and down-regulated SMAD4 expression. SMAD4 was highly expressed in HF cells, and silencing SMAD4 significantly decreased the α-SMA and Vimentin levels in HF cells. Collectively, the miR-324-3p may suppress the activation of HSCs and HF by targeting SMAD4. Therefore, miR-324-3p is identified as a potential and novel therapeutic target for HF.

## Introduction

Chronic liver diseases, such as liver cancer and cirrhosis, are the main causes of cancer-related deaths globally [1]. Chronic liver diseases occur because of many factors, including hepatic fibrosis (HF), which progressively develop to liver cancer and cirrhosis and severely affect patients’ health [2]. Several chronic disorders due to liver injury can result in HF, a condition caused by excessive tissue repair during the illness; these chronic disorders include alcoholic hepatitis (AH), chronic viral hepatitis, autoimmune liver diseases, as well as liver injury caused by metabolic diseases, parasitic infections, poisons, adverse drug reactions, etc. [3]. A characteristic of this process is the massive deposition of extracellular matrix (ECM) components, including fibrillar collagen, within the tissue [4, 5]. Although different causes of HF are associated with various biochemical and pathological processes, the activated hepatic stellate cells (HSCs) play an important role in HF [6, 7]. Progressive deposition of ECM components causes liver scarring because of HF [8, 9]. The activated stellate cells formed after liver injury undergo a significant phenotype alteration [10]. The activated HSCs produce the ECM and proliferate into fibers with HF progression, characterized by upregulated type I collagen. Besides, the activated HSCs produce other fibrosis-associated proteins [11]. In this process, fibrosis frequently activates HSCs as well. Such course is often accompanied with a high expression of Vimentin and α-smooth muscle actin (α-SMA). Recent research has implicated multiple intracellular signaling pathways in controlling HSC activation and HF progression [12, 13].

MicroRNAs (miRNAs), present in the human body, are small noncoding RNAs with 20–22 nucleotides in length. They regulate the expression of messenger RNAs (mRNAs) and bind to the 3ʹ- untranslated region (UTR) of the target gene. The degradation of mRNAs inhibits the synthesis of proteins [14]. The miRNAs exert vital functions in multiple biological processes, including cell proliferation. Inhibition of the posttranscriptional target gene expression can be achieved through linking with the coding regions, 3ʹ-UTRs, or 5ʹ-UTRs of the mRNA [15, 16]. Previous studies suggest that miRNA is abnormally expressed in diseases affecting the liver and other organs, such as fibrosis [17]. The regulation of miRNAs in liver diseases has been a hot research topic for the past several years. Many authors have demonstrated that miRNA-223 has crucial functions in liver inflammation as well as injury [18]. A related research has pointed out that miR-203 inhibits lipid accumulation in the liver and alcoholic fatty liver (AFL) progression [19]. Furthermore, miR-146a probably functions through negative feedback for the transforming growth factor (TGF)-β1 pathway in HF [20]. Besides, we also reviewed miRNAs’ role in HF with emphasis on interactions between miRNAs and mRNAs and the regulation of miRNAs. Although several miRNAs are dysregulated in HF, their molecular mechanism, expression profile, and biological function in HF, in particular HSCs, are unclear. To determine biomarkers linked with HF progression as well as pathological regression stages, the profiles of miRNA and mRNA expression in mice HSCs were analyzed. Notably, miR-324-3p is expressed, used, and metabolized in several cancer cells. A preliminary study by Chao Liu et al. showed that miR-324-3p regulated the invasion and migration of neural precursor cells (NPCs) by targeting WNT2B [21]. Hang Tuo et al. demonstrated that miR-324-3p promoted hepatocellular carcinoma (HCC) development by targeting DACT1 and activating the signaling of Wnt/β-catenin [22]. In gastric cancer cells, miR-324-3p activates Wnt/β-catenin signaling pathway by downregulating Smad4, according to Guang-Li Sun et al*.* [23]. During the early stages of our group, we performed complete transcriptome sequencing on primary HSCs of mice and thoroughly analyzed how circRNAs, miRNAs, and mRNAs are expressed in the cells. Our study involved the expression pattern, function, and mechanism of circFBXW4 in HF, and the assay outcomes suggested that miR-18b-3p, miR-324-3p and miR-18b-5p may directly bind to circFBXW4 [24]. It can be observed that circFBXW4 serves as a location for storing a variety of miRNA candidates linked to liver or fibrosis. Additionally, our group devoted considerable time to exploring the regulatory mechanism of non-coding RNA in liver fibrosis. In this study, we found that miR-324-3p had a significant impact on liver fibrosis. In contrast, the are few studies on miR-324-3p’s functions, expression, as well as mechanisms in HF. According to previous studies, miR-324-3p does not affect HSCs’ migration or invasion. Besides, it remains unknown whether miR-324-3p is a target for treating HF. Notably, HF is primarily caused by the TGF signaling pathway [25, 26]. The TGF-β superfamily’s intracellular signal transduction molecules are designated as SMADs. SMAD4 has an important function in TGF-β1 signaling according to multiple lines of evidence from biochemical and genetic studies. To be specific, SMAD4, chromosome 18q21, is a SMAD protein family member and mediates numerous benign diseases and malignant carcinomas [27]. Previous studies showed that SMAD4 plays a tumor suppressor role, and SMAD4 overexpression suppresses HCC cell invasion, proliferation, and migration [28]. In contrast, these results indicated that disrupting SMAD4 reduces the activity of promoters responding to SMAD3 in fibrosis. Through initiating the target gene transcription by SMAD3 regulation, SMAD4 plays a critical role in developing fibrosis disease [29]. Therefore, achieving a better understanding of HF’s molecular events is critical for preventing, diagnosing, and treating HF. The findings of this study revealed the molecular mechanisms through which HSCs were regulated by miR-324-3p under the conditions of in vitro and in vivo experiments; thus, a new biomarker with the potential to treat HF could be obtained.

## Materials and Methods

### Hepatic Fibrosis (HF) Mice Model Construction

All animals were purchased from the Experimental Animal Center of Anhui Medical University (No.81 Meishan Road, Shushan District, Hefei, Anhui Province, China). All animal experiments were approved by The Animal Experiment Ethics Committee of Anhui Medical University (LISC20190608). The mouse HF and HF recovery models was built as previously described [24].

**Fig. 1:**
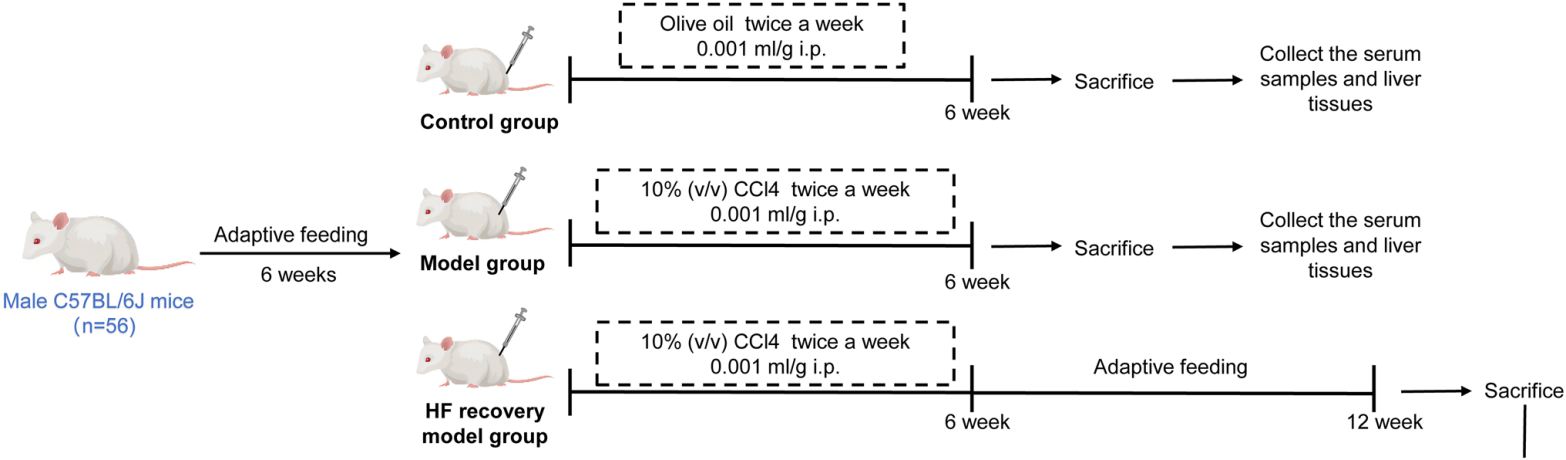
The schematic of the animal experiment

Fifty-six C57BL/6 J mice (male, 8–10 weeks, 22–25 g) was used in this study. All mice were kept at room temperature of 24 ± 1 °C and relative humidity of 40% to 70%, with free access to food, water and human care, while avoiding noise and keeping the environment clean. The mouse HF model was created using male C57BL/6 J mice after six weeks of adaptive feeding. The modeling and processing flow of mice is shown in Fig. 1. Briefly, HF mice were exposed to biweekly intraperitoneal injection of 10% (v/v) CCl_4_ at a dose of 0.001 ml/g [30]. For identical time intervals, the control mice were given identical amounts of olive oil as usual. Furthermore, the HF recovery model was established by injecting CCl_4_ at the same dose and frequency, but the injection was stopped after 6 weeks to avoid persistent chronic liver injury, followed by six weeks of normal feeding to trigger spontaneous reversion. After the final CCl_4_ injection, the animals were sacrificed two days later at the specified time. Then, the serum samples and liver tissues of mice were collected for histological, biochemical, and molecular analysis and subsequent research.

### Histopathology

To determine hepatic morphology and fibrosis in mice, hematoxylin and eosin (H&E) staining was performed on liver tissue. Firstly, the liver tissues were fixed with paraformaldehyde, embedded with paraffin, and sectioned into 6-µm-thick slices. The slices were dehydrated twice with xylene, rehydrated with gradient ethanol, and then subject to staining with H&E, Sirius red, and Masson’s trichrome [31]. Finally, the pathological changes in the tissues were evaluated and captured by microscopes.

### Isolation of Primary Hepatic Stellate Cells (HSCs)

Primary HSCs were obtained from mice as described previously [32]. Briefly, primary HSCs derived from mice liver were subjected to digestion with pronase E and collagenase IV (both: Sigma-Aldrich, USA) dissolved in PBS. Then, Nycodenz obtained from Axis-Shield Diagnostics (Norway) was used for layering suspensions of dispersed cells.

### Culture of Cells

LX-2 cells (human HSCs line) were obtained from the Type Culture Collection of the Chinese Academy of Sciences (Shanghai, China). LX-2 cell line was grown in Dulbecco's Modified Eagle Medium (DMEM) containing 10% Fetal Bovine Serum (FBS) and 1% penicillin/streptomycin (P/S] (Gibco, USA) [33]. Human TGF-β1 (5 ng/mL, R&D Systems, USA) was added to induce cell growth for 48 h. After incubation in 5% CO_2_ at 37 °C, the medium was refreshed at intervals of 24 h unless otherwise specified.

### Immunohistochemistry

The liver tissue was fixed in 10% neutral buffered formalin solution and embedded in paraffin for routine histology. The slides were de-paraffinized in xylene and dehydrated in alcohol, and the antigen extraction was achieved by microwaving in citric saline for 15 min. Then, the slides were covered with 3% H_2_O_2_ for 10 min to block endogenous peroxidase activity. After being blocked with 5% bovine serum albumin, the slides were incubated with primary antibody against α-SMA and SMAD4 overnight at 4 ℃. Upon rinsing, the sections were incubated with the corresponding secondary antibody for 1 h at room temperature. Next, antigenic sites were visualized by 3, 3′ -diaminobenzidine tetrahydrochloride (DAB) staining. After that, the slides were counterstained with hematoxylin for 1 minuted, dehydrated, and then observed using an Olympus microscope.

### Luciferase Reporter Assay

A reporter plasmid containing luciferase was packaged with the 3′ UTR sequence of SMAD4 mRNA. The specificity of miR-324-3p targeting SMAD4 mRNA was ascertained by co-transfection of psiCHECK2/has-miR-324-3p and SMAD4-Wt/has-miR-324-3p, SMAD4-Mut/has-miR-324-3p or the control vectors into 293 T cells using Lipofectamine 2000 (Invitrogen, USA). After 48 h of transfection, a dual-luciferase reporter assay kit (Promega, USA) was applied to determine the transfection results by the relative activity of firefly luciferase unit (RLU) according to the manufacturer’s instructions.

### Overexpression of miR-324-3p in Mice

The development and acquisition of adeno-associated virus (AAV) took place at Hanheng (Shanghai, China). One week before modeling mouse HF, AAV-miR-324-3p and vector (1 × 10^12^ VG/ml) diluted in saline were injected into the tail vein of mice, respectively. The mouse HF model was established by injecting CCl_4_ biweekly after one week of AAV administration. After establishing a mouse model of HF through chronic CCl_4_ intoxication for 6 weeks, the mice were anesthetized and sacrificed at the indicated time.

### Mimic and Inhibitors of miR-324-3p

LX-2 cells were transfected with Mimics and inhibitors of miR-324-3p (MK1910SL28, Biomics, China) using Lipofectamine 2000 reagent. Briefly, the cells were seeded into a 6-well plate containing DMEM, 1% P/S and 10% FBS for 24 h. Subsequently, cell transfection was performed with Lipofectamine 2000. Mimic and inhibitor of miR-324-3p were added to an Opti-MEM medium without antibiotics at final concentration of 40 nM and 80 nM, respectively (Gibco, USA). Upon 6 h of transfection, DMEM without antibiotics was used as the medium with TGF-β1 and 10% FBS. After transfection for 48 h, the cells were collected, and the total RNA and proteins were extracted.

### RNAi Analysis

Transient transfection of SMAD4 siRNA or a negative siRNA control (GenePharma, Shanghai) was performed in the cultured cells with Lipofectamine 2000 for 6 h to knockdown SMAD4. Subsequently, DMEM added with TGF-β1 was used as the medium. Incubation was performed under CO_2_ condition for 24 h at 37 °C, and the cells were then used for western blot assay.

### Flow Cytometry Analysis

As previously stated, the Cell Cycle and Apoptosis Analysis Kit (Beyotime, China) was used for cell cycle analysis in accordance with the manufacturer's protocol [31]. After transfecting LX-2 cells into 70% ethanol at 4 °C for 18 h, they were incubated with PE and detected using a CytoFLEX flow cytometer (Beckman Coulter, USA). Upon counting and comparison, the G0/G1 phase cell ratio was evaluated. Then, the apoptosis was detected using the Annexin V-FITC/PI Apoptosis Analysis Kit (Bestbio, China) and analyzed using a CytoFLEX flow cytometer (Beckman Coulter, USA). Besides, CytExpert software was employed for data analysis.

### Cell Counting Kit-8 (CCK-8) Assay

Based on the manufacturer's protocol, the proliferation of LX-2 cells was tested using cell counting kit-8 (CCK-8, Beyotime, China). Transfected LX-2 cells were seeded and treated with CCK-8 solution (10 μl) at 12th hour, 24the hour and 36th hour. Next, an Automatic Microplate Reader (BioTek, USA) was used to measure the optical density value at 450 nm after 2 h of incubation at 37 °C [34].

### Assay for Alanine Aminotransferase (ALT)/Aspartate Aminotransferase (AST) Activity

Referring to the protocol, serum alanine aminotransferase (ALT) and aspartate aminotransferase (AST) levels of mice were determined according to corresponding kits (Jiancheng, Nanjing, China). The absorbance values were estimated at 510 nm.

### Real-Time-Quantitative Polymerase Chain Reaction (RT-qPCR) and RNA Isolation

TRIzol reagent (Invitrogen, USA) was employed to extract total RNA from LX-2 cells, liver tissues and HSCs on the basis of the standard instructions. NanoDrop 2000 spectrophotometer (Thermo Scientific, USA) was used to estimate total RNA concentration [24]. Later, a Reverse Transcription Kit (Fermentas, USA) was adopted to synthesize complementary DNA (cDNA) from RNA. Next, real-time-quantitative polymerase chain reaction (RT-qPCR) of Vimentin, SMAD4, and α-SMA was conducted using SYBR-Green Master Mix Kit (TaKaRa, Japan) with RT-qPCR primers (Invitrogen, USA). Then, miR-324-3p was detected by one-step miRNA RT-qPCR (Biomics, China). Besides, glyceraldehyde-3-phosphate dehydrogenase (GAPDH) and β-actin were used to normalize the mRNA ratios. The primer sequences are shown in Table 1.

**Table 1:** Primers sequences used for real-time PCR

Genes	Forward primer (5’-3’)	Reverse primer (5’-3’)
*Mouse*		
GAPDH	GGACCTCATGGCCTACATGG	TAGGGCCTCTCTTGCTCAGT
α-SMA	GTCCCAGACATCAGGGAGTAA	TCGGATACTTCAGCGTCAGGA
Vimentin	CGAAAACACCCTGCAATCTT	GTGAGGTCAGGCTTGGAAAC
*Human*		
β-actin	GGCATTCACGAGACCACCTAC	CGACATGACGTTGTTGGCATAC
α-SMA	GTGTTGCCCCTGAAGAGCAT	GCTGGGACATTGAAAGTCTCA
Vimentin	CGA AAACACCCTGCAATCTT	GTGAGGTCAGGCTTG GAA AC

### Western Blot (WB) Assay

Total protein was extracted from the collected mice liver tissues and LX-2 cells with the standardized method using radio-immunoprecipitation assay (RIPA) lysis buffer (Beyotime, China) containing 1% phenylmethanesulfonyl fluoride (PMSF) [35]. Subsequently, the supernatant was collected after 30 min of centrifugation at 10,000 g at 4 °C. The proteins levels were estimated using the NanoDrop 2000 spectrophotometer. Protein was separated through 12% sodium dodecyl sulphate–polyacrylamide gel electrophoresis (SDS-PAGE), then transferred onto polyvinylidene fluoride (PVDF) membranes (Millipore Corp, USA). The membranes were blocked for 1–2 h with 5% nonfat milk and then washed three times with tris buffer solution (TBS)-tween20 buffer for 10 min each. Next, the membranes were incubated with specific primary antibodies overnight. The primary antibodies used are as follows: anti-SMAD4 (Abcam, China; 1:1000), anti-β-actin (Bioss, China; 1:500), anti-SMA (Bioss; 1:500), and anti-Vimentin (Bioss, 1:500). After washing with TBS-Tween20 buffer for three times, horseradish peroxidase (HRP)-conjugated anti-rabbit (Bioss, China) were used as secondary antibody (1:10000) to treat the membranes at room temperature for 1 h after 3 times of washing with TBS/Tween20 (0.075%). Subsequently, an enhanced chemiluminescence system (Bio-Rad, USA) was used for visualizing the bands. Lastly, immunoblotting images were subjected to quantitative densitometric analyses by ImageJ software (NIH, USA).

### Statistical Analysis

The data were expressed as mean ± standard error of measurement (SEM) and analyzed by one-way analysis of variance (ANOVA) and the post hoc test using Newman–Keuls method. GraphPad Prism 5.0 (GraphPad, USA) was responsible for statistical analysis. *P* < 0.05 was considered statistically significant.

## Results

### MiR-324-3p Decreases in Hepatic Fibrosis (HF) But Recovers in HF Recovery Model

**Fig. 2:**
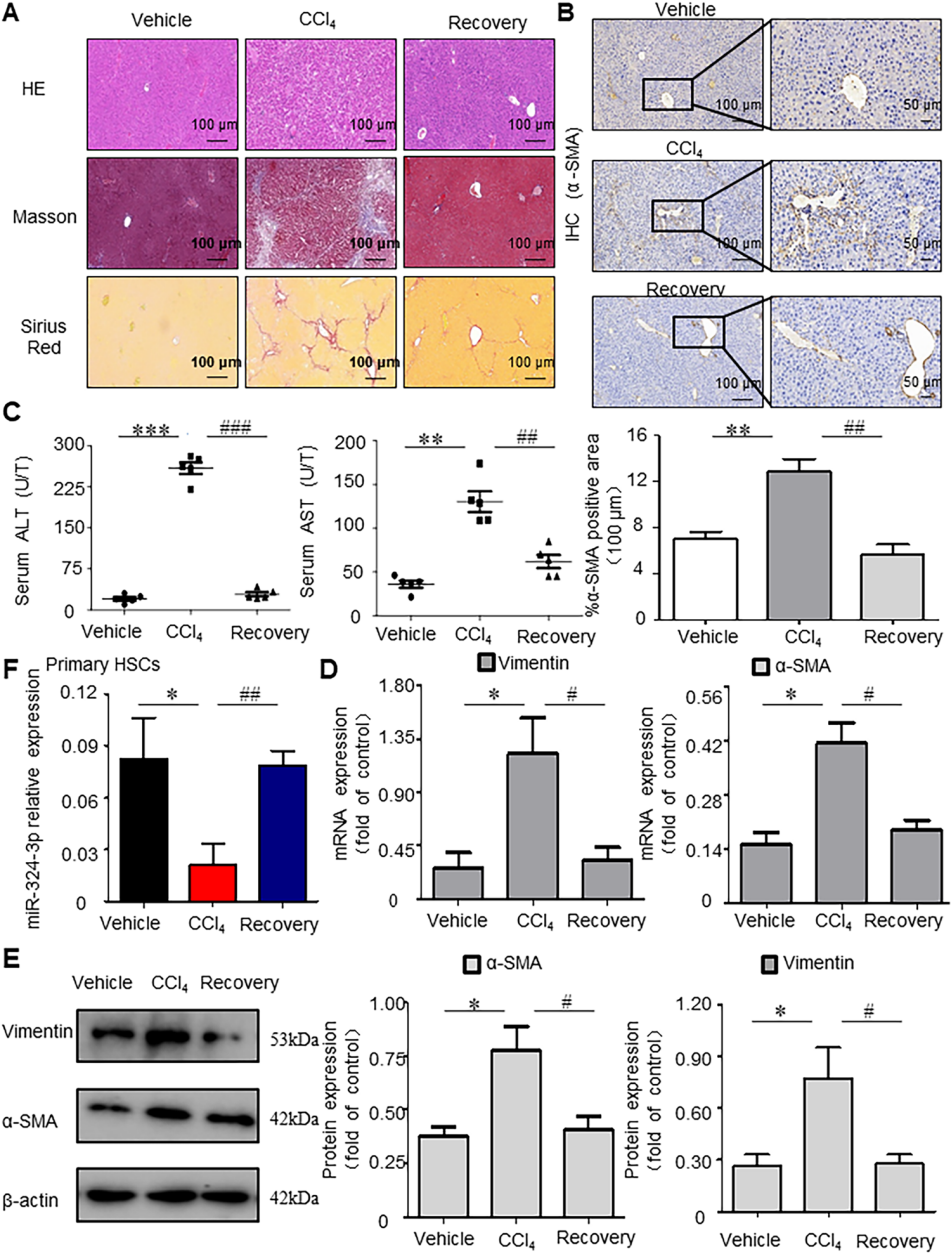
miR-324-3p decreases in hepatic fibrosis (HF) but restores in HF recovery. **A** Staining of liver tissues with hematoxylin and eosin (H&E), Masson’s trichrome, and Sirius red. Scale bar, 100 μm. **B** Immunohistochemical (IHC) staining results of α-SMA in the liver tissues. Scale bars: 100 and 50 μm. Ipwin32 software was used to measure the positive staining areas. **C** Measurement of serum alanine aminotransferase (ALT) and aspartate aminotransferase (AST) levels. **D** Real-time-quantitative polymerase chain reaction (RT-qPCR) to detect Vimentin and α-SMA mRNA levels. **E** Western blot (WB) assay for protein expression levels of α-SMA and Vimentin. **F** In one-step RT-qPCR, the miR-324-3p expression in HF mice was compared to that of normal mice. ^*^P < 0.05, ^**^P < 0.01, ^***^P < 0.001 *vs*. Control group; ^#^*P* < 0.05, ^##^*P* < 0.01, ^##^*P* < 0.001 *vs*. Recovery group

In order to explore the role and mechanism of miR-324-3p in HF, the HF mouse model was induced by CCl_4_ and the HF injury recovery model was established by reversing repeated injury and self-recovery. In addition, the HF recovery model was established by injecting CCl_4_ at the same dose and frequency, but the injection was stopped after 4 weeks to avoid persistent chronic liver injury, followed by 6 weeks of normal feeding to trigger spontaneous reversion. Subsequently, the pathological characteristics in mice were first evaluated. In the histological examination, H&E, Masson’ s trichrome and Sirius red staining results showed that CCl_4_-treated mice had a liver injury and increased collagen deposition, and the injury of liver tissues were alleviated in HF recovery mice (Fig. 2A). Furthermore, α-SMA immunostaining and serum ALT, AST levels were obviously increased in CCl_4_-treated mice compared with those in Control group, while they were lower in Recovery group than in CCl_4_ group (*P* < 0.01, Fig. 2B, C). Similarly, WB and RT-qPCR analysis revealed that the mRNA and protein expression levels of α-SMA and Vimentin were remarkably upregulated in HF mice compared with those in Control group. Interestingly, the mRNA and protein expression levels of Vimentin and α-SMA were restored during recovery (*P* < 0.05, Fig. 2D, E). The findings above indicated that CCl_4_-induced HF mice model was successfully established. In addition, miR-324-3p was downregulated in mouse HF tissues in comparison to that in Control tissues as determined by RT-qPCR. On the contrary, miR-324-3p was upregulated in liver tissues during HF recovery (*P* < 0.05, Fig. 2F).

### Lower miR-324-3p Expression of Transforming Growth Factor (TGF)-β1-Activated LX-2 Cells In Vitro

**Fig. 3:**
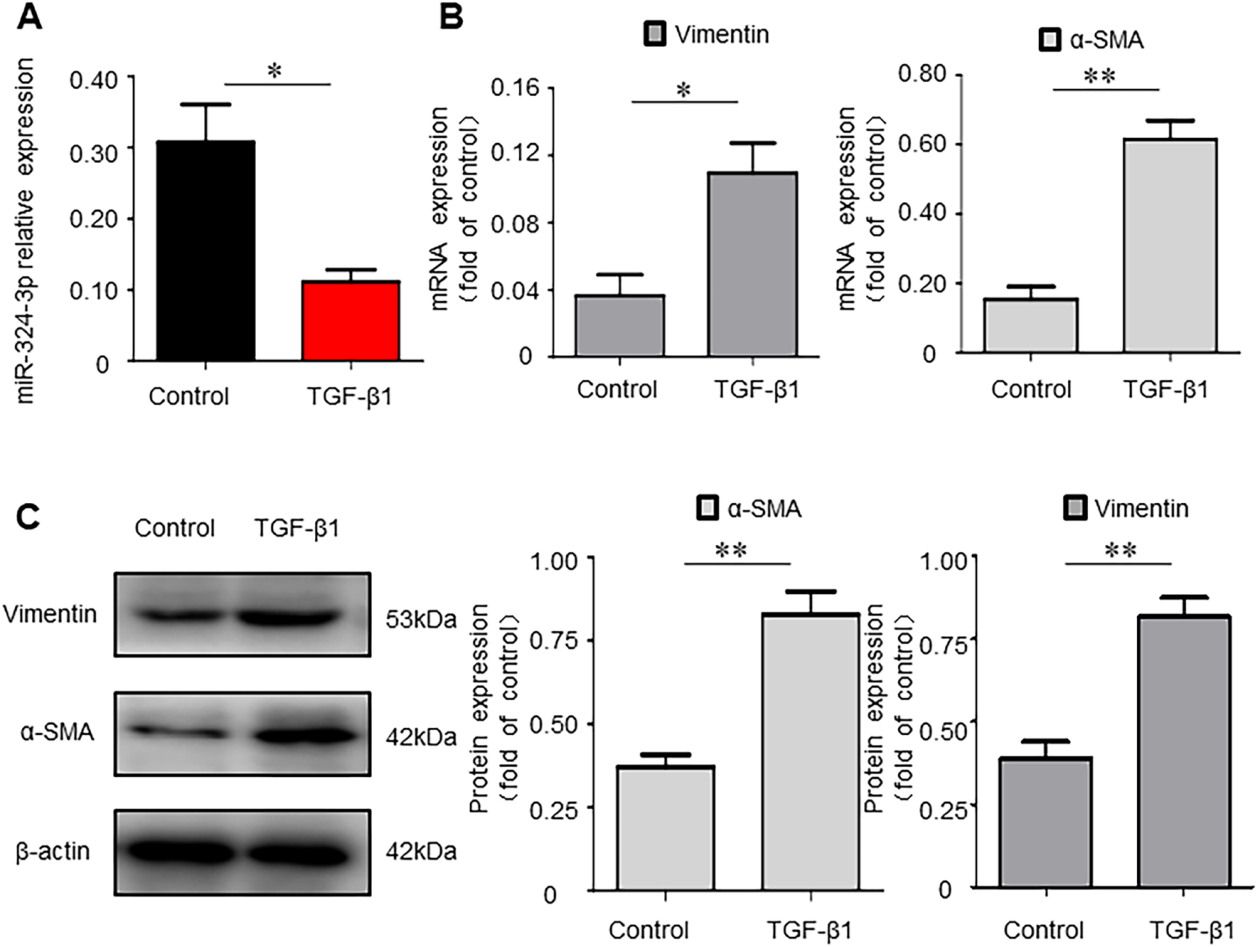
Lower miR-324-3p expression of transforming growth factor (TGF)-β1-activated LX-2 cells in vitro. **A** One-step real-time-quantitative polymerase chain reaction (RT-qPCR) to assess the miR-324-3p expression in LX-2 cell line. **B** Both smooth muscle actin (SMA) and Vimentin mRNA levels were measured through RT-qPCR. **C** Western blot (WB) assay to test α-SMA and Vimentin protein expression. ^*^P < 0.05 and ^**^P < 0.01 *vs*. Control group

In the TGF-β1-activated LX-2 cells, miR-324-3p level was remarkably decreased compared with that in Control group (*P* < 0.05, Fig. 3A). Moreover, fibrogenic genes α-SMA and Vimentin showed significant increased mRNA expression levels in TGF-β1-activated LX-2 cells compared with in Control group (*P* < 0.05, Fig. 3B). WB results also demonstrated that α-SMA and Vimentin were upregulated in TGF-β1 group as compared to Control group (*P* < 0.01, Fig. 3C). These data confirmed our earlier finding that miR-324-3p expression decreased in HF cell model.

### Antifibrotic Effects of miR-324-3p in Hepatic Fibrosis (HF) Mice In Vivo

**Fig. 4:**
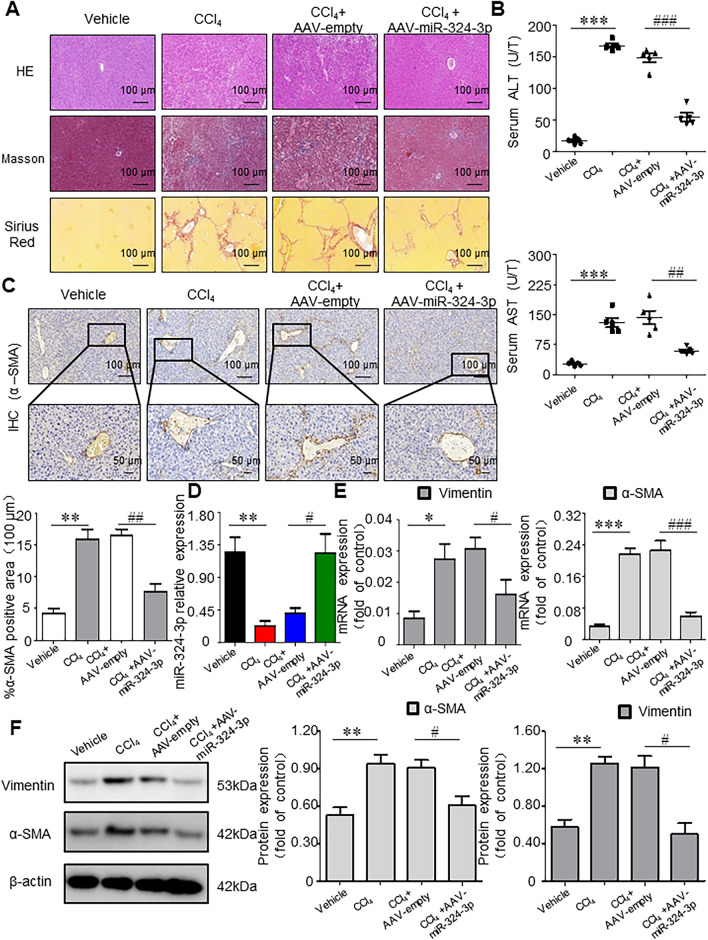
Antifibrotic effects of miR-324-3p on hepatic fibrosis (HF) mice in vivo. **A** Hepatic tissues stained by Masson, hematoxylin and eosin (H&E), and Sirius red stains for pathological observation. Scale bar, 100 cm. **B** Measurement of alanine aminotransferase (ALT) and aspartate aminotransferase (AST) levels in serum. **C** immunohistochemical staining for α-smooth muscle actin (α-SMA) in tissues of the liver. Scale bars: 100 and 50 μm. Ipwin32 software was used to measure the positive staining areas. **D** Expression of the miR-324-3p was confirmed through real-time-quantitative polymerase chain reaction (RT-qPCR). **E**, **F** WB assay as well as RT-qPCR for protein and mRNA expression of fibrogenic genes. ^*^P < 0.05, ^**^P < 0.01, ^***^P < 0.001 *vs*. Control group; ^#^*P* < 0.05, ^##^*P* < 0.01, ^##^*P* < 0.001 *vs*. CCl_4_ + AAV-empty group

Histological assessment showed that collagen deposition and liver injury in HF mice were reduced after AAV-miR-324-3p treatment compared with AAV-empty treatment (Fig. 4A). HF mice infected with AAV-miR-324-3p showed significantly decreased serum ALT and AST levels compared with HF mice infected by AAV-empty (Fig. 4B). Moreover, immunohistochemistry showed a marked decline in α-SMA staining in liver tissues following AAV-miR-324-3p administration compared to AAV-empty administration (Fig. 4C). Subsequently, an AAV9 vector was administered systemically to CCl_4_-induced mice to overexpress the miR-324-3p (Fig. 4D). Compared with AAV-empty-infected HF mice, Vimentin and α-SMA protein and mRNA levels were reduced in the AAV-miR-324-3p-infected HF mice (Fig. 4E, F). However, there were no significant differences in miR-324-3p expression, pathological tissue injury, serum ALT, serum AST, α-SMA expression and Vimentin expression between HF group (CCl_4_ group) and HF (CCl_4_) + AAV-empty group. Overall, the miR-324-3p was markedly downregulated in liver fibrogenesis, and miR-324-3p overexpression mediated by the AAV9 vector ameliorated liver injury and downregulated myofibroblast marker.

### MiR-324-3p Suppresses the Activation of Transforming Growth Factor (TGF)-β1-Induced LX-2 Cells In Vitro

**Fig. 5:**
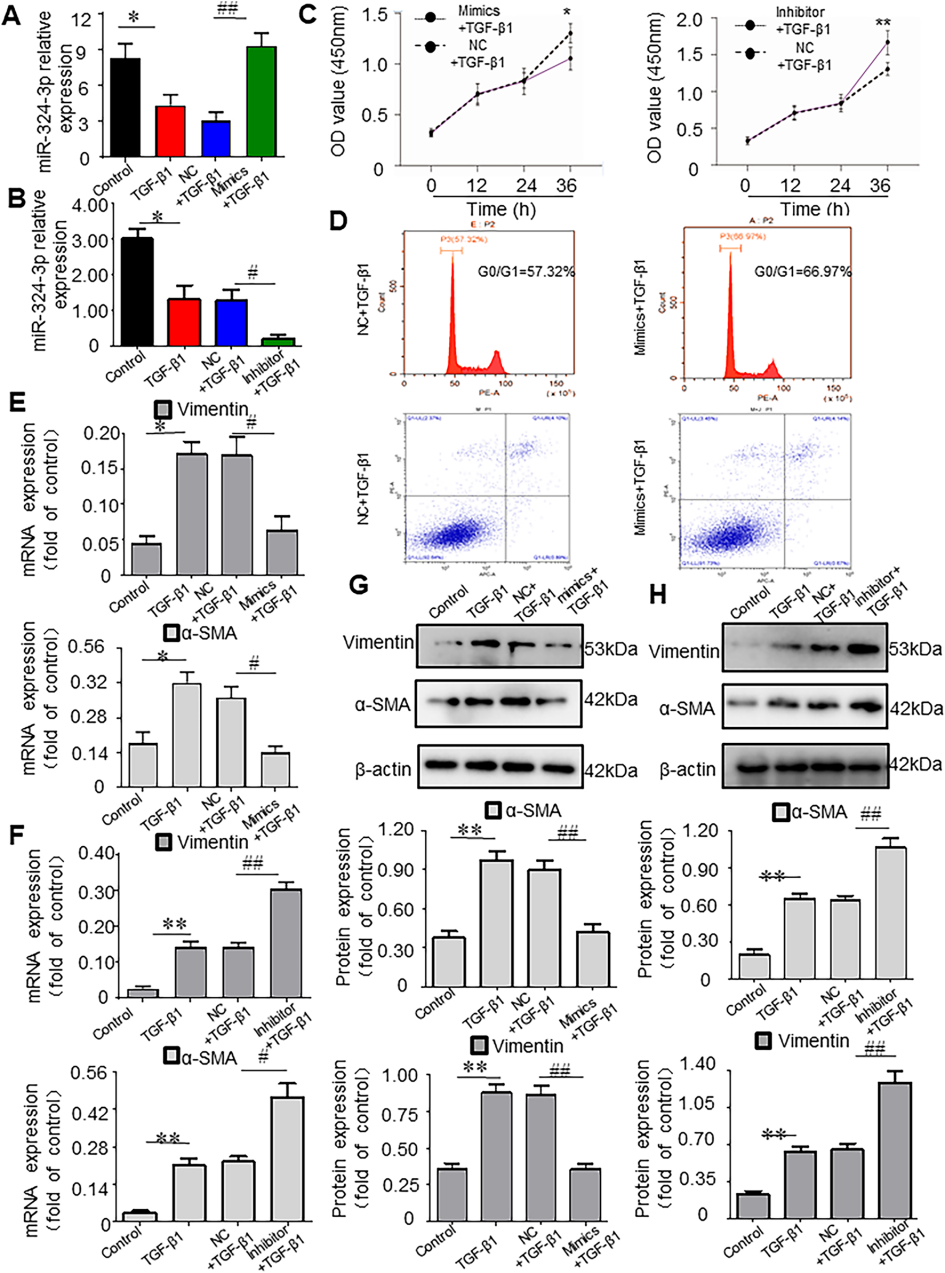
MiR-324-3p suppresses the activation of transforming growth factor (TGF)-β1- induced LX-2 cells in vitro. **A** Hepatic stellate cells (HSCs) were transfected with miR-324-3p mimic, and expression of the miR-324-3p was determined by real-time-quantitative polymerase chain reaction (RT-qPCR). **B** RT-qPCR to analyze the miR-324-3p expression in HSCs transfected with the miR-324-3p inhibitor. **C** Cell counting kit-8 (CCK-8) to analyze the proliferation of transforming growth factor (TGF)-β1-induced LX-2 cells with indicated treatment. **D** Flow cytometry to detect the cycle and apoptosis of TGF-β1-induced LX-2 cells. **E**, **G** Western blot (WB) assay and RT-qPCR to evaluate α-smooth muscle actin (α-SMA) and Vimentin expression in transfected HSC cells. **F**, **H** The α-SMA, and Vimentin levels in transfected cells were analyzed by WB assay as well as RT-qPCR. ^*^P < 0.05 and ^**^P < 0.01 *vs*. Control group; ^#^*P* < 0.05 and ^##^*P* < 0.01 *vs*. NC + TGF-β1 group

To assess the effect of miR-324-3p on TGF-β1-induced LX-2 cells (a HSC line of human origin having critical characteristics of activated HSCs), miR-324-3p in TGF-β1-induced LX-2 cells was overexpressed and knocked down by transfecting with miR-324-3p mimics and inhibitors, respectively (Fig. 5A/B). According to the assay results, TGF-β1-induced HSC cells transfected with miR-324-3p mimics had a significantly lower proliferation capacity than those transfected with NC (*P* < 0.05); while TGF-β1-induced HSC cells transfected with miR-324-3p inhibitor showed much higher proliferation capacity than those transfected with NC (*P* < 0.01) (Fig. 5C). Further, flow cytometry showed that overexpression of miR-324-3p blocked the cell cycle in G1 phase. However, there was no significant difference in the number of apoptotic cells (Fig. 5D). Enhancement of miR-324-3p remarkably decreased the α-SMA and Vimentin mRNA and protein expression in TGF-β1-induced LX-2 cells. Conversely, miR-324-3p knockdown enhanced α-SMA and Vimentin mRNA and protein levels (Fig. 5E–H).

### MiR-324-3p Directly Regulates SMAD4 in Hepatic Fibrosis (HF) and Hepatic Stellate Cells (HSCs)

**Fig. 6:**
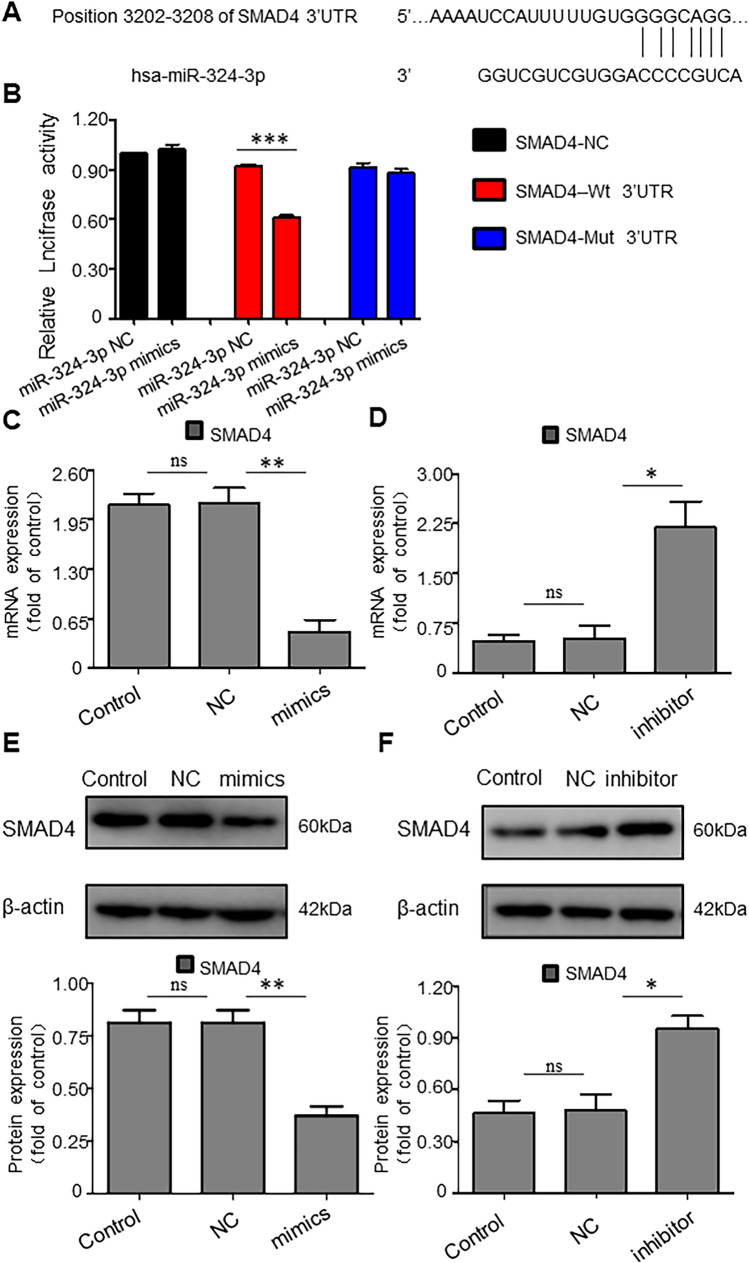
miR-324-3p regulates SMAD4 expression directly in hepatic fibrosis (HF) and hepatic stellate cells (HSCs). **A** The miR-324-3p seed sequence bound to SMAD4 mRNA's 3'- untranslated region (UTR) based on bioinformatics analyses. **B** Luciferase reporter gene method to verify the direct targeting relationship between miR-324-3p and SMAD4, ^***^P < 0.001. **C**, **D** SMAD4 mRNA levels were analyzed after transfection with mimics through real-time-quantitative polymerase chain reaction (RT-qPCR). (E, F) SMAD4 protein expression was measured by western blot (WB) assay after transfection of mimics. ^ns^*P* > 0.05 *vs*. Control group. ^*^P < 0.05 and ^**^P < 0.01 *vs*. NC group

The miRNAs were proved to suppress translation and induce mRNA degradation by binding to 3ʹ-UTR or 5ʹ-UTR of target mRNAs. Then, miR-324-3p target genes were searched using miRBase and TargetScan. As shown in Fig. 6A, SMAD4 3ʹ-UTR contained miR-324-3p’s putative binding sites. To verify the targeting relationship between miR-324-3p and SMAD4, we cloned SMAD4 NC or SMAD4-Wt 3 '-UTR or SMAD4-Mut 3' -UTR into luciferase expression vectors and co-transfected 293T cells with miR-324-3p NC or miR-324-3p mimics. The results showed that the luciferase activity in 293T cells with co-transmutation SMAD4-Wt 3 '-UTR and miR-324-3p mimics was significantly lower than that in other groups (*P* < 0.001, Fig. 6B). In our experiments, SMAD4 mRNA and protein expression levels were reduced in miR-324-3p mimic-transfected HSCs (Fig. 6C, E), while SMAD4 mRNA and protein levels were increased in the inhibitor-transfected HSCs (Fig. 6D, F).

**Fig. 7:**
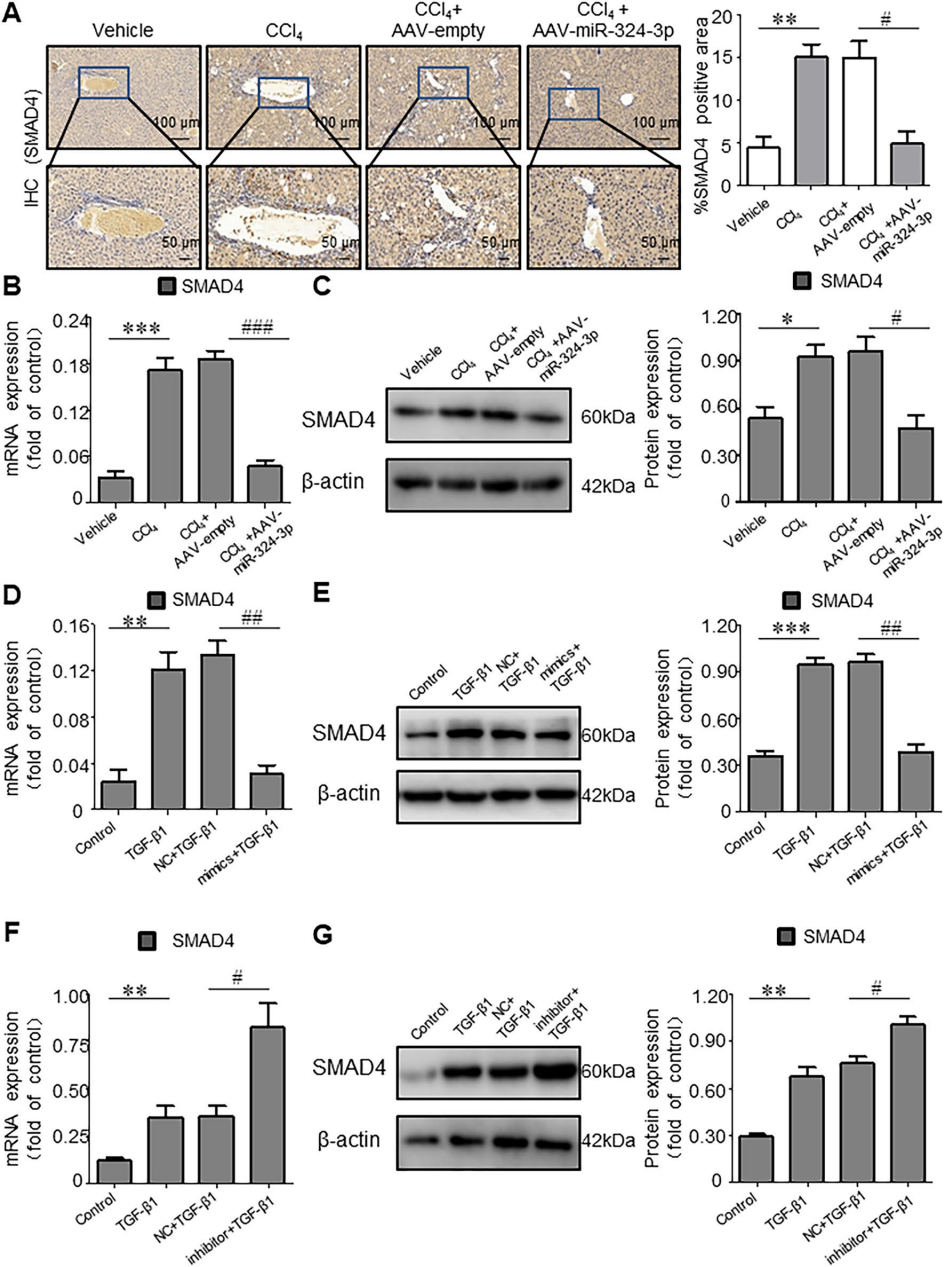
miR-324-3p regulates SMAD4 in hepatic fibrosis (HF) and hepatic stellate cells (HSCs). **A** Four groups were compared by immunohistochemical (IHC) analysis of liver tissues. **B** Real-time-quantitative polymerase chain reaction (RT-qPCR)-based determination of SMAD4 expression in liver tissues. **C** SMAD4 expression in mice liver tissues was analyzed by western blot (WB) assay. **D**, **E** SMAD4 expression was assessed by WB assay and RT-qPCR after cell transfection with the mimic or NC and treated with transforming growth factor (TGF)-β1 for 24 h. **F**, **G** An inhibitor or NC was transfected into the cells, and the cells were then stimulated with TGF-β1 for 24 h. After that, the WB assay and RT-qPCR were adopted for measuring SMAD4 expression. ^*^P < 0.05, ^**^P < 0.01, ^***^P < 0.001 *vs*. Control group; ^#^*P* < 0.05, ^##^*P* < 0.01, ^##^*P* < 0.001 *vs*. NC group

Subsequently, the targeted regulatory effect of miR-324-3p on SMAD4 was further explored through HF models in vivo and in vitro. RT-qPCR and IHC results showed that the expression level of SMAD4 in liver tissues of CCl_4_-induced HF mice was significantly higher than that in Control mice; and overexpression of miR-324-3p through transfection of AAV-miR-324-3p could significantly reduce the expression of SMAD4 in liver tissues of CCl_4_-induced HF mice (Fig. 7A, B, C). As shown in Fig. 7D, E, F, and G, the mRNA and protein expression levels of SMAD4 in TGF-β1-induced LX-2 cells were significantly higher than those in Control group. In addition, relative to transfection with NC mimics (TGF-β1 + NC group), transfection with miR-324-3p mimics significantly decreased the SMAD4 mRNA and protein expression levels in TGF-β1-induced LX-2 cells. On the contrary, transfection with miR-324-3p inhibitor significantly increased the expression of SMAD4 in TGF-β1-induced LX-2 cells.

### SMAD4 Enhances Myofibroblast Marker Expression in Transforming Growth Factor (TGF)-β1-Induced LX-2 Cells In Vitro

**Fig. 8:**
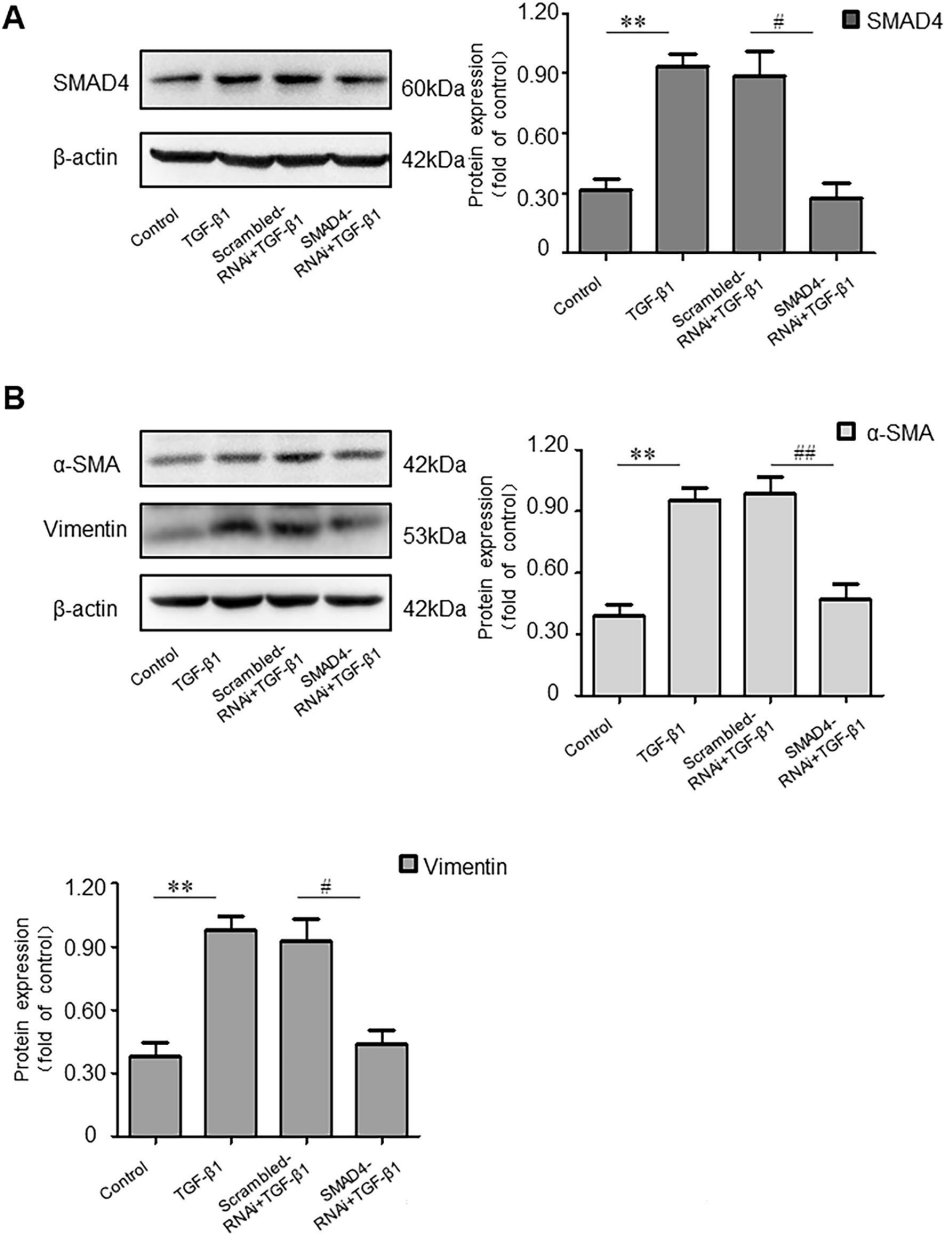
SMAD4 enhances myofibroblast marker expression in transforming growth factor (TGF)-β1-induced LX-2 cells in vitro. **A**, **B** SMAD4 protein expression was measured by western blot (WB) assay after transfection of Scrambled RNAi or SMAD4-RNAi and treated with transforming growth factor (TGF)-β1 for 24 h. ^**^P < 0.01 *vs*. Control group. ^#^*P* < 0.05 and ^##^*P* < 0.01 *vs*. Scrambled RNAi + TGF-β1 group

To clarify the role of SMAD4 in HF, SMAD4 was silenced in TGF-β1-induced LX-2 cells via siRNA. WB results revealed that siRNA-SMAD4 successfully inhibited SMAD4 protein expression in TGF-β1-induced LX-2 cells (Fig. 8A). Furthermore, α-SMA and Vimentin protein levels were increased after siRNA-SMAD4 treatment in TGF-β1-induced LX-2 cells (Fig. 8B). These results suggested that SMAD4 played a promoting role in HF.

## Discussion

Among persons aged 45 to 54 years and 55 to 64 years, chronic liver disease and cirrhosis are the 4th and 7th leading cause of death, respectively. But based on accumulated evidence, the mortality due to liver ailments has been underestimated consistently over the past few decades [36]. HF is manifested as a wound-healing response in almost all chronic liver diseases, and during this process, hepatic parenchyma accumulates and forms scar tissue. The predominant cell type responsible for fibrogenesis is HSCs. In response to inflammatory stimuli or hepatocyte death, HSCs undergo trans-differentiation to myofibroblast-like cells [37]. HSC proliferation leads to ECM accumulation through collagen secretion, which forms fibrous scar tissues beneficial for initiating the repair of tissues. Nevertheless, HSC proliferation causes cirrhosis if not correctly controlled [38]. TGF-β1, as a major profibrogenic liver cytokine, can indirectly organize α-SMA stress fibers and induce collagen I production by activating quiescent HSCs. Hence, finding new targets to treat liver disease is very critical. Therapeutic agents can be developed by controlling the pathways and factors that lead to fibrosis [39].

Based on the latest research progression, miRNAs exert critical functions in HF. Previous studies have linked various stages of HF to dysregulated miRNAs and HSC activation [40–42]. For instance, the miR-324-3p is linked to chronic hepatitis and HCC [43]. However, the functions and relevant mechanisms of miR-324-3p in HF remain to be investigated. Here, we assessed how miR-324-3p affected HF and HSCs here. In our study, HF mice showed a remarkable downregulation in miR-324-3p compared with the control mice; while the miR-324-3p levels were restored in HF recovery mice. Additionally, HSC activation was significantly inhibited, myofibroblast transdifferentiation was decreased, injury of liver fibrogenesis was minimized, deposition of collagen was reduced, and fibrogenic factors were inhibited by miR-324-3p overexpression. Moreover, we found that miR-324-3p played an anti-fibrosis role by blocking the cell cycle of HSCs and inhibiting cell proliferation. These findings indicated that miR-324-3p contributed to stopping the progression of HF. Hence, we suggest that miR-324-3p has antifibrotic effects on HF and can be considered a biomarker to treat HF. In addition, α-SMA and Vimentin gene expression levels were inhibited in the activated LX-2 cell line expressing miR-324-3p. Furthermore, miR-324-3p overexpression suppressed cell proliferation and activation. Overall, miR-324-3p participates in activating HSCs and ameliorating HF pathogenesis in mice.

Mechanistically, miR-324-3p acts as a modulator by targeting various mRNAs in liver diseases [44]. SMAD4 is a SMAD family member and proved to play a role in HCC development [45]. TGF-β functions biologically by binding to its serine/threonine kinase-mediated receptors (TGF-βRI and TGF-βRII), and it then triggers signaling pathways through SMADs, the downstream mediators/transcription factors. The phosphorylation of TGF-βRI by type II receptor kinases leads to SMAD3 and SMAD2 phosphorylation through the activated type I receptors, which then trigger the binding of the SMAD4 helper proteins. Following the translocation into the nucleus, the SMAD complex binds to specific promoters and activates the process of HF. Therefore, as downstream TGF-β signaling transcription factors, SMADs might also be therapeutic targets for preventing and treating HF in addition to preventing and treating liver cirrhosis and HCC. Thus, SMADs could be assumed as targets to control the stimulation of HSCs in HF. In particular, SMAD4 expression decreases following miR-324-3p overexpression in liver fibrogenesis as miR-324-3p binds to SMAD4 3ʹ-UTR. Based on our results, miR-324-3p is a critical factor in HF development as miR-324-3p upregulation inhibits LX-2 cells’ activation and proliferation.

Nevertheless, there are some possible limitations in our study. For example, the research basis of this study stemmed from the derived miR-324-3p from mouse sequencing results. LX-2 cells are commonly utilized as tool cells in liver fibrosis research [46]. In this study, LX-2 cells were selected to improve clinical transformation in the later stage. To enrich and perfect our research, other cells, such as HSC-T6 cells, will be employed for experiments in the future.

## Conclusion

**Fig. 9:**
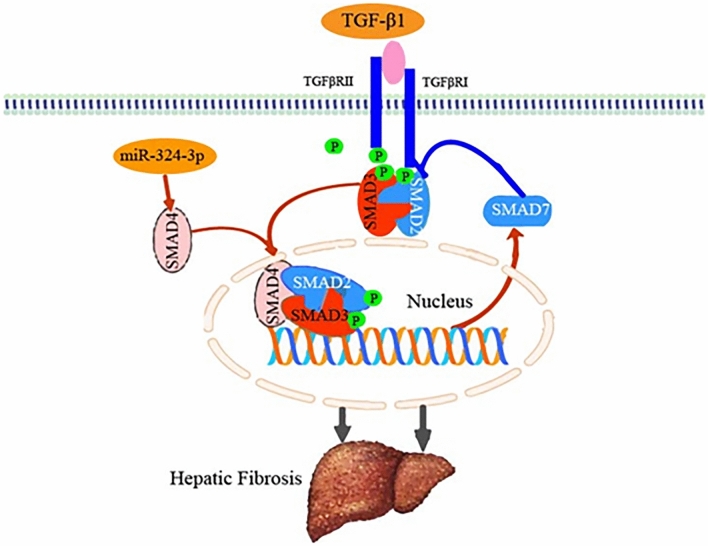
The central mechanism by which miR-324-3p exerts its effects on hepatic fibrosis (HF). The mechanisms by which miR-324-3p regulated HF were shown in a schematic diagram. Transforming growth factor (TGF)-β1 treatment attenuated the miR-324-3p expression level, which enabled direct binding to SMAD4 and subsequent suppression of HF. SMAD4 was not only the target gene of miR-324-3p, but also played a role in liver fibrosis

To sum up, we identified that miR-324-3p acted as an inhibitor of liver fibrosis and was down-regulated in CCl_4_-induced HF mice liver tissues and TGF-β1-induced HF cells. Highly expressed miR-324-3p can significantly inhibit liver tissue damage and decrease serum ALT and AST levels in HF mice, down-regulate the fibrosis-related biomarkers expression in HF cells and mice tissues, and then alleviate liver fibrosis. Such effects of miR-324-3p may be achieved by down-regulating SMAD4. In addition, we also proposed that SMAD4 exerted a double function in HF (Fig. 9). SMAD4 is not only the target gene of miR-324-3p, but also plays a role in liver fibrosis. Our present data suggest that restoring miR-324-3p expression can provide a new potential marker for treating HF.

## Data Availability

Upon reasonable request, the corresponding author can provide the data used to support the study findings.
